# Culturable Screening of Plant Growth-Promoting and Biocontrol Bacteria in the Rhizosphere and Phyllosphere of Wild Rice

**DOI:** 10.3390/microorganisms10071468

**Published:** 2022-07-20

**Authors:** Zongmu Yao, Yalin Chen, Shouyang Luo, Jilin Wang, Jiafan Zhang, Jianfeng Zhang, Chunjie Tian, Lei Tian

**Affiliations:** 1 Key Laboratory of Mollisols Agroecology, Northeast Institute of Geography and Agroecology, Chinese Academy of Sciences, Changchun 130102, China; amuu0316@gmail.com (Z.Y.); yalin12383@126.com (Y.C.); shouyang8253@163.com (S.L.); jiafan1415@126.com (J.Z.); tiancj@iga.ac.cn (C.T.); 2 Key Laboratory of Straw Biology and Utilization, The Ministry of Education, College of Life Sciences, Jilin Agricultural University, Changchun 130118, China; zhangjianfeng06@tsinghua.org.cn; 3 Rice Research Institute, Jiangxi Academy of Agricultural Sciences, National Engineering Research Center for Rice, Nanchang 330200, China; wangjilin1982@163.com

**Keywords:** wild rice, rhizosphere microorganisms, biocontrol bacteria, growth-promoting bacteria

## Abstract

Wild rice is an important improved resource for cultivated rice and its unique ability to resist biotic and abiotic stress has attracted the attention of many scholars. The microbial community structure in the rhizosphere and leaf area of different rice varieties is also different, which may be one of the reasons for the difference in stress resistance between wild rice and cultivated rice. Forty-six bacteria were screened from the rhizosphere and phyllospheric of four different wild rice varieties. The results of functions of the screened strains showed that 18 strains had a good inhibitory effect on rice blast, and 33 strains had the ability to dissolve phosphorus, potassium, or fix nitrogen. Through potted experiment, the three bacterial strains, 499G2 (*Peribacillus simplex*), 499G3 (*Bacillus velezensis*), and 499G4 (*B. megaterium*) have a positive effect on the growth of cultivated rice in addition to the resistance to rice blast. The contents of total nitrogen, total phosphorus, total potassium, indole acetic acid (IAA), and chlorophyll in plant leaves were increased. In addition, in the verification test of rice blast infection, the application of inoculants can significantly reduce the content of malondialdehyde (MDA), increase the content of soluble sugar, and increase the activity of plant antioxidant enzymes, which may thereby improve rice in resisting to rice blast.

## 1. Introduction

Rice occupies an important position in the world’s food crops, and about 50% of the populations live on rice as the staple food [1]. Rice blast is an important rice disease caused by the fungus *Magnaporthe grisea* and is considered to be one of the most important diseases in rice planting due to its worldwide distribution and severe yield loss [2]. Rice blast can cause necrotic lesions of leaves and panicles. Panicle blast is more damaging than leaf blast because it causes greater rice yield losses [3]. There are more resources for resistance to rice blast in wild rice [4]. Its disease and pest resistance genes are often used for the cultivation of cultivated rice. For example, the rice disease resistance gene Pi33 was introduced from wild rice [5]. The aggregation of Pi54/Piz-t, Pi35/Pi21, and Pigm/Pi37 can make a rice variety have broad-spectrum resistance to the rice blast pathogen [6,7].

Due to the relative ease of quantifying microorganisms based on metagenomics and next-generation sequencing, there has been increased interest in the properties of soil-associated microbiota [8]. Root deposition and exudates can affect the composition of the microbial community around the rhizosphere, and microbial activity can affect plant growth and health. Different host plant genotypes lead to differences in these microbial communities [9,10]. The effects of domestication and breeding on plant physiology and development may have a large impact on the microhabitats inhabited by root-associated microorganisms. During the process of domestication, fundamental changes have taken place in plant structure. During the transition from wild species to modern cultivars, some desirable microbes, such as rhizospheres, which are beneficial to plant stress resistance, may be lost. Such changes may affect microbial populations associated with rice roots through selection for traits such as drought avoidance, water tolerance, or yield. Numerous studies have found that microorganisms such as endophytes, arbuscular mycorrhizal fungi (AMFs), rhizobia, etc. are also important in the evolution of their host plant species [11]. These microbes have tight interactions with their host plants. Some of these microorganisms can improve plant stress resistance, especially in wild species, through interactions with host plants [11,12,13]. The study by Shenton [14] showed that the root-associated bacterial community have small but significant differences among rice genotypes [15]. Differences among bacteria associated with different plant genotypes were only weakly related to the phylogenetic distance among cultivated rice varieties [9,16]. This suggests that root traits selected during domestication may have a significant impact on rhizosphere microbiota composition. Researchers find stronger relationship between fungi and bacteria in cultivated crops compared to wild relatives [17]. Related studies have also shown that wild varieties have higher beneficial symbiont colonization and lower pathogen presence compared to cultivated varieties [18,19]. The results showed that compared with cultivated varieties, wild varieties had higher abundance of beneficial symbiotic bacteria and lower abundance of pathogens. Crop domestication affected fungal communities more than bacterial communities and improved microbial relationships in the rhizosphere of cultivated crops [17]. Similarly, with the help of high-throughput sequencing, researchers found that rice cultivation had a profound effect on rice rhizosphere microbial communities because the rhizosphere microbial community of cultivated rice in agro-ecosystems is more sensitive to environmental changes than that of wild rice in natural ecosystems [20]. It is reasonable to assume that the rhizosphere microbiome of wild crops may be more effective than their cultivated relatives in promoting the growth and survival of host plants under biotic and abiotic stress conditions [2,8,9].

Based on the above description, we make the following hypothesis: (1) part of the resistance of wild rice to rice blast is due to its rhizosphere microorganisms, and applying it to cultivated rice can improve the resistance of cultivated rice to rice blast; (2) the perennial characteristics of wild rice enable it to recruit more growth-promoting bacteria to provide nutrients for it, and the application of wild rice rhizosphere and phyllosphere bacteria to cultivated rice can promote the growth of cultivated rice.

## 2. Materials and Methods

### 2.1. Experiment Material

Four wild rice varieties and two cultivated rice varieties were used in the experiment: Nivara wild rice (*Oryza nivara*), Medicinal wild rice (*O. officinalis* Wall. ex Watt), common wild rice (*O. rufipogon* Griff.), spotted wild rice (*O. punctata*), Meitezhen indica rice (Oryza *sativa* Linn. subsp. indica Kato), and Daohuaxiang japonica rice (*O. sativa* L. subsp. japonica) was provided by the Northeast Institute of Geography and Agroecology, Chinese Academy of Sciences.

### 2.2. Screening of Culturable Bacteria in Rhizosphere and Phyllosphere

Different wild rice roots and leaves collected from potted plants were rinsed with distilled water, placed in a sterilized petri dish, and soaked in alcohol for 30 s. During this period, the surface was sterilized by flipping with tweezers, and then rinsed with distilled water to remove the surface alcohol. Then the samples were ground in a mortar and transferred to a 10 mL tube. Then, the same amount of distilled water is added into the tubes to dilute the grinding liquid. Then, extract 200 μL of the mixture and place it in a solid LB medium (NaCl 10 g, tryptone 10 g, yeast powder 5 g, agar 18 g, distilled water 1 L) for culture spread evenly in the dish. After three days, the culture condition was observed, and strains with different shapes were selected for isolation and purification.

### 2.3. Identification of Strains

For bacteria, the 16S rRNA gene was selected for detection (the sequences of the paired primers are 27F 5′-AGAGTTTGATCCTGGCTCAG-3′ and 1492R 5′-TACGACTTAACCCCAATCGC-3′). The reaction system is: 2× TaqPCR Master Mix 12.5 μL, 1 μL of forward primer and reverse primers, ddH_2_O 9.5 μL. Reaction procedure: 95 °C for 5 min; 94 °C for 1 min, 55–58 °C for 1 min, 72 °C for 90 s, 30 cycles; 72 °C for 10 min. After electrophoresis, obvious bands can be observed, and they are sent to the testing center (Sangon Biotech, Shanghai, China) for bacterial species identification.

### 2.4. Assessment of Antagonistic Effect

The rice blast pathogen was placed in the center of a petri dish containing PDA medium (potato 200 g, glucose 20 g, distilled water 1 L, and agar 18 g) in the form of a cake, and the bacteria were inoculated at a distance of 20 mm around the center for a confrontation experiment. Another culture dish that was not inoculated with bacteria and only inoculated with rice blast pathogen was used as a control. After 15 days of culture, the length and width of pathogenic bacteria colonies were measured and the inhibition rate of antagonistic bacteria against pathogenic fungi was calculated. Bacteriostasis = (D − B)/d (100%) (D: CK pathogenic bacteria colony diameter; B: treatment pathogenic bacteria colony width), repeat each treatment for 3 times, take only the pathogenic bacteria cake as the control (CK), and take the average of 3 repetitions. In addition, in order to distinguish the effect of bacterial cells or bacterial fermentation broth on rice blast pathogenic bacteria, the bacteria with antagonistic effect were subjected to liquid fermentation for 1 day and the supernatant after centrifugation was filtered through a filter membrane; 50 μL was taken instead of bacterial cells for confrontation experiment.

### 2.5. Evaluation of Bacteria Growth-Promoting Activity

The screened bacteria were inoculated into NBRIP medium (glucose 10 g, (NH_4_)_2_SO₄ 0.5 g manganese sulfate 0.03 g, magnesium sulfate 0.3 g, sodium chloride 0.3 g, ferrous sulfate 0.03 g, potassium chloride 0.3 g, tricalcium sulfate 5 g, and distilled water 1 L), Ashwagandha medium (potassium dihydrogen phosphate 0.2 g, magnesium sulfate 0.2 g, sodium chloride 0.2 g, glucose 10 g, calcium carbonate 5 g, and calcium sulfate 0.1 g), potassium solution medium (glucose 10 g, MgSO_4_ 0.2 g, dipotassium hydrogen phosphate 0.2 g, 1% ferric chloride 2 drops, ammonium sulfate 0.5 g, sodium chloride 0.2 g, agar 18 g, potassium feldspar powder 5 g, calcium sulfate 0.2 g, calcium carbonate 5 g, and distilled water 1 L) to observe the growth, and after 7 days of culture, when the strain has the corresponding growth-promoting effect, it will produce a transparent circle in the screening medium. We determined the effect according to the growth of bacteria in each petri dish and the size of the transparent circle: able to grow in selective medium (+), the transparent circle is the same size as the colony (++), the transparent circle is slightly larger than the colony (+++), the transparent circle is the size of the colony more than 2 times (++++).

The method of bacterial starch hydrolysis ability used Dong’s method [21]. Bacteria were cultured on LB medium containing 0.2% for 2 days and then Lugol’s iodine solution was added dropwise to observe the medium. If there was a transparent circle around the bacteria, it is positive, otherwise it is negative.

Determination of bacterial IAA used the Salkowsky method [22]. The bacterial strains were cultured in LB shake flasks supplemented with L-tryptophan (100 μg/mL) for 48 h, the culture was centrifuged at 10,000× *g* for 15 min, and the supernatant was collected. Exactly 2 mL of Salkowsky reagent (add 1 mL of 0.5 M FeCl_3_ to 50 mL of 35% HClO_4_) and 1 mL of supernatant were reacted at 28 ± 2 °C for 30 min. After the end, the absorbance was measured with a spectrophotometer at 535 nm. It was pink, indicating the presence of indole acetic acid (IAA). A standard curve was produced by dissolving different known concentration of IAA in LB medium containing Salkowsky reagent. The IAA (μg/mL) in the culture filtrate was quantified.

### 2.6. Pot Experiment

For pot experiment, 100 rice seeds were selected and then rinsed with distilled water and soaked in 70% alcohol for 3 min for surface disinfection. Then, the seeds were rinsed 3 times with distilled water and immersed in 5% sodium hypochlorite solution for 30 s, then we washed away residual sodium hypochlorite with sodium thiosulfate. The seeds were placed in a sterile petri dish, immersed in distilled water, wrapped in black plastic film, and subjected to shading treatment for 24 h. Afterwards, the seeds were moved to a petri dish containing moist filter paper for 72 h and the germinated seeds were moved to a seedling tray for 10 days. The seedlings with the same growth and development were transplanted into flower pots, 3 plants in each pot, and sprayed with different bacterial liquids. A second spraying was carried out after 20 days and after 30 days. Afterwards, the growth indexes and physiological and biochemical indexes of the plant were measured.

The rice was taken out of the pots and we measured the length of the shoot and the width of the leaf with a ruler. We weighed with a ten thousand balance and calculated the root-to-shoot ratio.

A plant’s total nitrogen and total phosphorus were measured by continuous flow analyzer and total potassium was measured by inductively coupled plasma emission spectroscopy. Chlorophyll was determined by ethanol extraction [23]. Leaf IAA yield was carried out using Zhang’s method [24].

To test the effects of the inoculation of the screened bacteria and *M. grisea* on the content of MDA, Pro, SS, SOD, and POD in rice leaves, 8 treatment CK (blank control), CK + PoC (rice blast control), 499G2 + PoC (499G2 and rice blast together application), 499G3 + PoC (499G3 bacteria and rice blast were applied together), 499G4 + PoC (499G4 bacteria and rice blast were applied together), 499G2 (499G2 bacteria were applied alone), 499G3 (499G3 bacteria were applied alone), and 499G4 (499G4 bacteria were applied alone agent). The pots test method is the same as above. The rice blast was evenly sprayed on the surface of the leaves. After 12 h of shading and moisturizing with a black plastic bag, the concentration of 3 different biocontrol agents (499G2, 499G3, and 499G4) was diluted to 10^7^ and sprayed evenly on the surface of the leaves. The unsprayed pot was used as a negative control, and the rice without inoculation of inoculum and rice blast was used as a positive control.

The infection of rice was detected after 7 days of rice blast infection, and the content of malondialdehyde (MDA) [24], proline (Pro) [24], Superoxide dismutase (SOD), Peroxidase (POD) [25], and soluble sugar (SS) [24] content of cultivated rice were measured. The above results were statistically analyzed by ANOVA test with SPSS software with *p* value < 0.05.

## 3. Results

### 3.1. Screening and Identification of Culturable Bacteria in the Rhizosphere and Phyllosphere of Rice

A total of 45 bacterial colonies with different morphology were screened from the rhizosphere and phyllosphere of the four different wild rice varieties. The screening sites and identification results are shown in Table 1, including *Bacillus amyloliquefaciens* (348Y4), *B. anthracis* (348G3, 499G6), *B. arbutinivorans* (218Y4), *B. aryabhattai* (218G4), *B. cereus* (218G3), *B. firmus* (499Y7), (218G2, 315G4), *B. licheniformis* (348Y5), *B. megaterium* (348Y6, 315G5, and 499G4), *B. methylotrophicus* (315G6), *B. muralis* (315Y6), *B. mycoides* (315G1), *B. oryzaecorticis* (218Y2), *B. pseudomycoides* (315G3), *B. simplex* (315Y4, 499Y1), *B. stratosphericus* (499Y3), *B. subtilis* (348Y3, 218Y3, 218G6, and 499Y5), *B. thioparans* (315Y5), *B. velezensis* (348Y1, 348Y2, 315Y1, 315Y3, 315Y7, 315G2, and 499G3), *Brevibacterium frigoritolerans* (348G2), *Enterobacter asburiae* (348G1, 348G4, and 315Y2), *Fictibacillus nanhaiensis* (348Y7), *F. phosphorivorans* (499Y6 and 499G7), *Lysinibacillus fusiformis* (218Y1), *Pantoea septica* (218G1), *Peribacillus simplex* (499G2), *Priestia megaterium* (499G5), and *Pseudomonas frederiksbergensis* (218G7).

**Figure 1:**
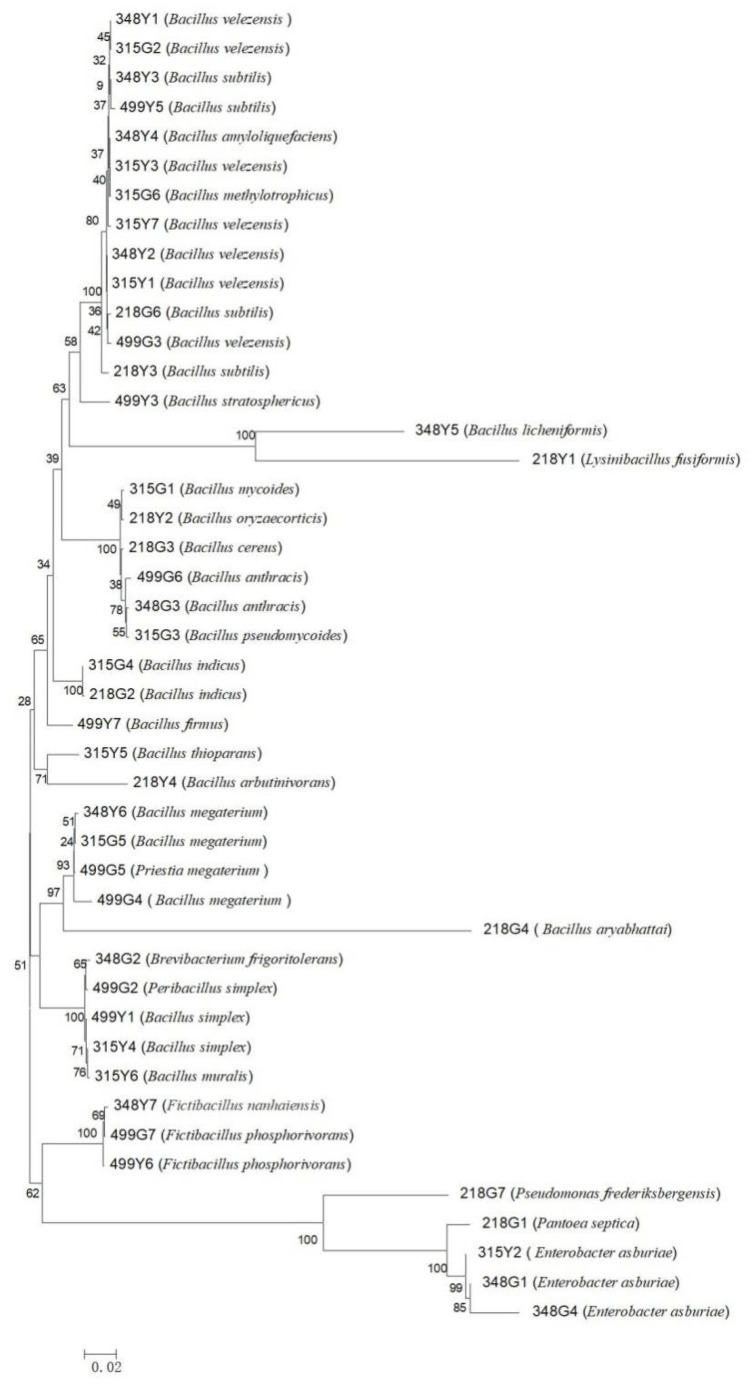
Phylogenetic tree diagram of 45 bacterial strains.

Through the physiological and biochemical identification of bacteria, it can be seen that 27 bacteria have the effect of amylase (348Y1, 348Y2, 348Y3, 348Y4, 348Y5, 348G1, 348G3, 315Y1, 315Y2, 315Y3, 315Y6, 315G1, 315G2, 315G3, 315G5, 218Y1, 218Y2, 218Y3, 218G3, 218G6, 499Y5, 499G2, 499G3, 499G4, 499G5, 499G6, and 499G7), 11 strains of bacteria capable of producing IAA (348Y6, 348G1, 315Y2, 315Y4, 315G5, 218G1, 218G4, 499Y6, 499G3, 499G4, and 499G7). Among them, 218G4 has the strongest ability of IAA production, reaching 121.55 μg/mL. Based on the phylogenetic tree of the screened 45 bacteria, it can be seen that the rhizosphere and phyllosphere bacteria of the 4 wild rice species have certain similarities, but generally, they are quite different (Figure 1).

### 3.2. Assessment of Antagonistic Effect

**Figure 2:**
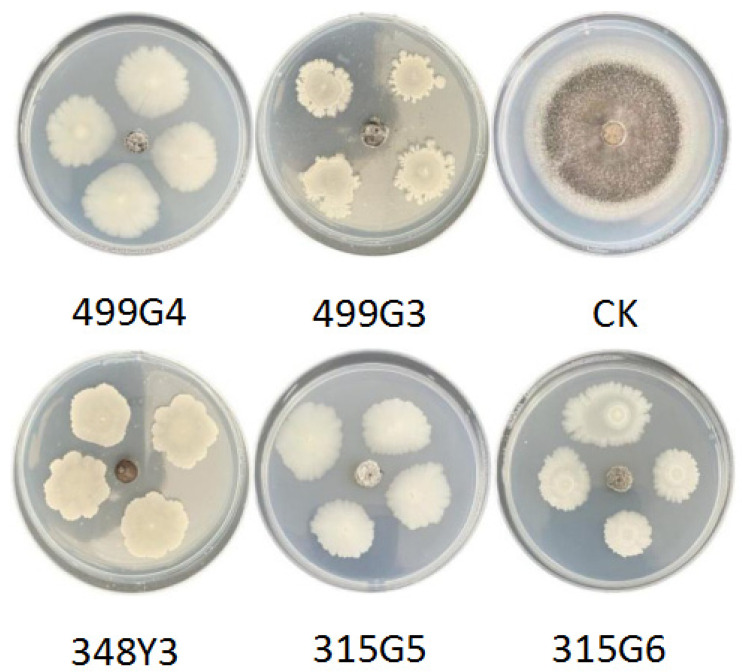
Antagonistic effect of the screened bacteria 499G4, 499G3, 348Y3, 315G5 and 315G6 on *Magnaporthe grisea*.

The screened strains were inoculated on the PDA medium with the pie of pathogenic strain of *M. grisea* to carry out the antagonistic effect of the strains. Results verified that a total of 18 bacteria had antagonistic activity against the rice blast pathogen. After 15 days of growth, the CK treatment of the rice blast pathogen could basically cover the entire PDA plate. However, rice blast after inoculation with wild rice rhizosphere and phyllosphere bacteria received a significant inhibitory effect (Figure 2). The inhibition rate of 17 strains of bacteria (499Y3, 218G6, 218Y3, 315G1, 315G2, 315Y1, 499G3, 218G1, 499Y5, 315Y3, 315Y7, 348Y1, 348Y3, 348Y4, 499G2, 499G4, 315G5, and 315G6) against rice blast can reach more than 80% (Table 2). Among them, the inhibition effect of 499G4 is the most obvious, reaching 87.31%. By comparing the identification results of bacterial species, it can be seen that the two screened genera, *B. velezensis* and *B. subtilis,* functioned best in preventing rice blast and occupy a large proportion. After the fermentation broth antagonism experiment, the growth of blast disease was not restricted. It can be seen that the inhibitory effect of these 18 bacterial strains on rice blast was mainly bacterial inhibition directly.

### 3.3. Evaluation of Bacteria Growth-Promoting Activity

After screening and identification of selective medium, a total of 29 bacteria (218G1, 218G2, 218G4, 218G7, 218Y3, 315G1, 315G2, 315G5, 315Y1, 315Y2, 315Y4, 315Y6, 315Y7, 348G1, 348G2, 348Y1, 348Y4, 348Y5, 348Y6, 348Y7, 499G1, 499G3, 499G4, 499G5, 499G7, and 499Y1) have the ability to dissolve phosphorus, potassium solution, or fix nitrogen (Table 3). Among them, three strains (218G1, 218G4 and 348G1) can simultaneously fix nitrogen, dissolve phosphorus, and dissolve potassium. There are five strains of bacteria (348Y7, 315Y7, 315G5, 218Y3, and 348Y5) that can dissolve both phosphorus and potassium and 218G7 has the functions of nitrogen fixation and potassium solution. 315Y2 bacteria have nitrogen-fixing and phosphorus-dissolving functions. The ability is divided into four levels according to the colony growth and the size of the transparent circle: able to grow in selective medium (+), the transparent circle is the same size as the colony (++), and the transparent circle is slightly larger than the colony (+++). The transparent circle is more than two times of the size of the colony (++++). Experiments found that most of the bacteria screened out can convert potassium feldspar into potassium, which will help plant in absorbing and utilizing solid potassium. Among them, 499G5 and 315Y5 have the most prominent abilities, but their dissolving effect on tricalcium phosphate is not obvious. Even though it can be grown without available phosphorus, it exhibits smaller transparent circles.

### 3.4. Results in Pot Experiment

**Figure 3:**
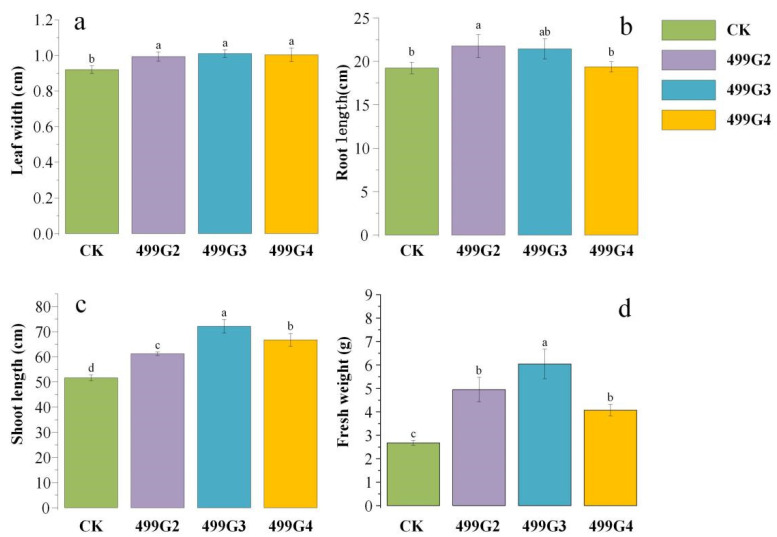
Effects of 499G2, 499G3 and 499G4 on leaf width (**a**), root length (**b**), shoot (**c**) and fresh weight (**d**) of rice. Error bars represent standard errors (*n* = 3). Different lowercase letters show statistically significant difference (*p* value < 0.05).

Exactly 18 strains of bacteria that have antagonistic effects against the rice blast pathogen were subjected to potted growth-promoting experiments and phenotypic observation found that 11 of them had a growth-promoting effect on rice. A number of three rice plants with the most obvious effect were selected for the determination of physiological and biochemical indicators. It can be seen that the width of rice leaves increased after the application of inoculants (Figure S1). Compared with the control, the width of rice leaves after 499G2, 499G3 and 499G4 treatments increased by 7.97%, 9.78%, and 9.06% (Figure 3a). The increase of leaf width is more conducive to the photosynthesis and respiration of rice and the growth-promoting effect of 499G2 inoculum on rice root length is more obvious, which is increased by 13.17% compared with the control (Figure 3b). The application of 499G3 and 499G4 had no significant effect on rice root development. The three inoculants all significantly increased the shoot length of rice, among which 499G3 had the most obvious effect, which could increase the shoot length by 39.61%. As seen in Figure 3c, 499G2 and 499G4 increased by 18.51% and 25.16%. In addition, 499G3 also significantly increased the fresh weight of rice by 125.79% (Figure 3d). This is closely related to its promotion of aboveground growth.

**Figure 4:**
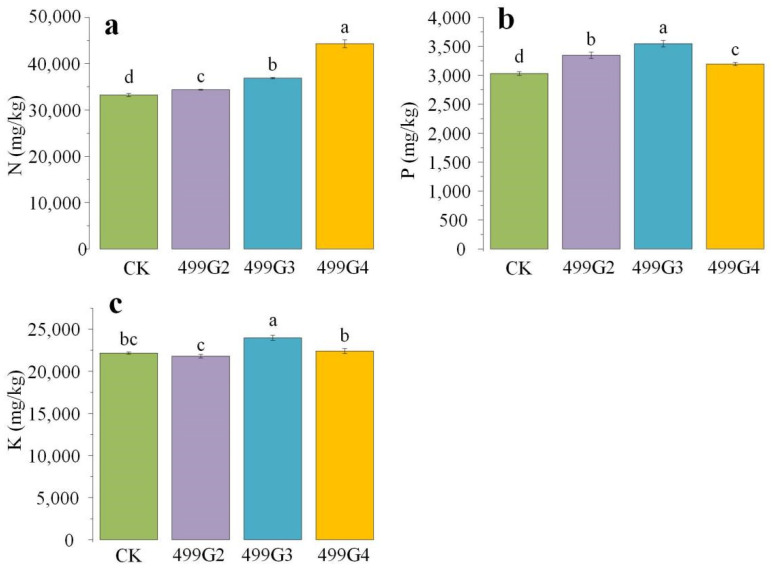
Effects of inoculum application on total nitrogen (**a**), total phosphorus (**b**) and total potassium (**c**) contents in cultivated rice. Error bars represent standard errors (*n* = 3). Different lowercase letters show statistically significant difference (*p* value < 0.05).

From the effect of inoculants on the nitrogen, phosphorus, and potassium content of plants, it can be seen that 499G4 can effectively increase the total nitrogen level of plants, which is 33.33% higher than that of the control (Figure 4a). Observing the changes in phosphorus content, it was found that 499G2, 499G3, and 499G4 could significantly increase the phosphorus content in plant leaves, which were increased by 10.44%, 17.01%, and 5.45%, respectively, compared with the control group (Figure 4b). Comparing the total potassium content in leaves under different inoculant treatments, only 499G3 had a significant effect on the increase of potassium, reaching 8.17% (Figure 4c), while 499G2 and 499G4 did not change much compared to the control group.

**Figure 5:**
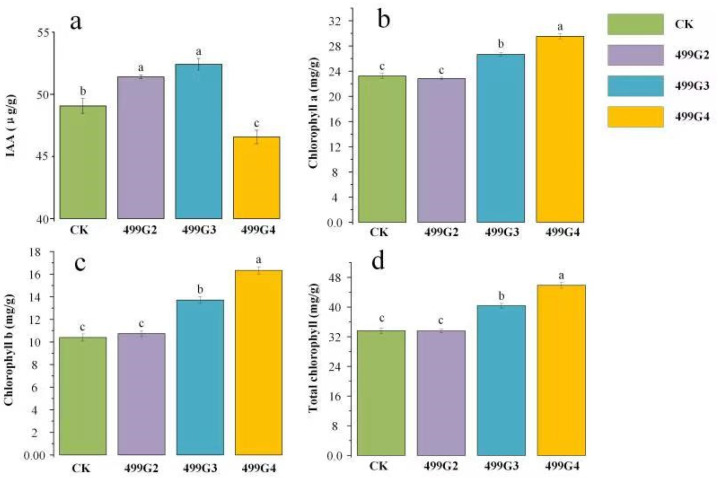
Effects of inoculants on IAA (**a**), chlorophyll a (**b**), chlorophyll b (**c**) and total chlorophyll (**d**) in cultivated rice. Error bars represent standard errors (*n* = 3). Different lowercase letters show statistically significant difference (*p* value < 0.05).

From the accumulation of IAA and the content of chlorophyll, it can be seen that the content of IAA in rice plants after the application of 499G2 and 499G3 was significantly increased, which increased by 4.90% and 6.82%, respectively, compared with the control (Figure 5a). However, the application of 499G4 resulted in a decrease in IAA, as the plant growth was much higher than that of the control. Therefore, we tested the chlorophyll of the plants and found that the application of 499G4 can significantly increase the content of chlorophyll a, chlorophyll b, and total chlorophyll compared with the control (Figure 5b–d). Among them, the increase of chlorophyll b was the most significant, reaching 56.92%. 499G3 increased chlorophyll a, chlorophyll b, and total chlorophyll by 13.60%, 31.92%, and 20.24%, respectively, compared with the control, while 499G2 had no significant effect on plant photosynthesis.

### 3.5. Effect of Bacterial Application on Rice Resistance to Magnaporthe grisea

**Figure 6:**
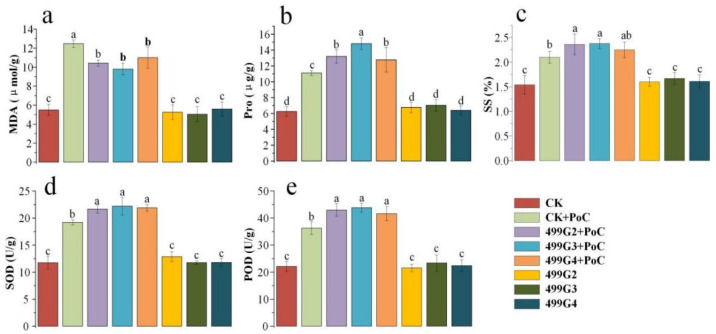
Effects of the inoculation of the screened bacteria and *M. grisea* on the content of MDA (**a**), Pro (**b**), SS (**c**), SOD (**d**) and POD (**e**) in rice leaves. CK, blank control; CK + PoC, rice blast control; 499G2 + PoC, 499G2 and rice blast together application; 499G3 + PoC, 499G3 bacteria and rice blast were applied together; 499G4 + PoC, 499G4 bacteria and rice blast were applied together; 499G2, 499G2 bacteria were applied alone; 499G3, 499G3 bacteria were applied alone; and 499G4, 499G4 bacteria were applied alone agent. Error bars represent standard errors (*n* = 3). Different lowercase letters show statistically significant difference (*p* value < 0.05).

By measuring MDA, Pro, SS, SOD, and POD of rice leaves, the physiological state of control check (CK), only *M. grisea* (pots of control, PoC), inoculant + PoC, and inoculant treated plants was determined. Obviously, the activity of MDA became higher after the infection of rice blast fungus. Compared with uninfected leaves, the content of MDA increased by 126.67% and the content of MDA could be significantly reduced after inoculation (Figure 6a), compared with the control inoculated with *M. oryzae*, the MDA content decreased by 16.31%, 21.39%, and 11.76% after inoculation with 499G2, 499G3 and 499G4, respectively.

Proline is an osmotic regulation substance secreted by plants under stress, and its content reflects the ability of plants to deal with environmental changes. The experimental data show that the content of proline in each treatment group was significantly increased after inoculation with *M. oryzae* (Figure 6b). The inoculant can increase the accumulation of proline in rice leaves again and the effect of 499G3 + PoC is the most significant (33.43%), followed by 499G2 + PoC (18.95%), 499G2 + PoC (18.95%), and 499G4 + PoC (15.05%). The change of soluble sugar content was similar to that of proline. With the infection of rice blast, the soluble sugar content of each group increased significantly (Figure 6c), but the increase was different. Ratio has the highest soluble sugar increase (13.51%), followed by 499G2 + PoC (12.56%). It can be observed that 499G3 + PoC also helped to increase the soluble sugar, but the effect is not obvious compared with CK + PoC.

After 10 days of rice blast infection, the activities of peroxidase (POD) and superoxide dismutase (SOD) in each group were measured, and inoculation of inoculants in the state of rice blast infection could significantly improve leaf cell defense activity. The increase of SOD activity was (12.92–15.73%), and the increase of POD activity was (14.69–20.66%) (Figure 6d,e).

Through the determination of MDA, Pro, SS, SOD, and POD in rice leaves inoculated with microbial inoculum, it can be seen that although the stress resistance index of plants after inoculation with microbial inoculum fluctuates, the change is not significant compared with that of normal growing rice.

## 4. Discussion

### 4.1. Screening and Identification of Biocontrol Bacteria

Wild rice is an important resource for the improvement of cultivated rice, and the role of its rhizosphere makes wild rice have higher stress resistance. To perform the rice blast antagonism experiment, we screened 45 bacterial strains from the rhizosphere and phyllosphere of the 4 different varieties of wild rice. Among them, 18 strains had significant antagonistic effect, and the inhibiting rate of 17 strains reached more than 80%. In addition, through the identification of the growth-promoting ability of these 45 bacteria, it was found that 12 bacteria have the ability to produce IAA, and 27 bacteria can hydrolyze amylase. In the pot experiment of 18 biocontrol bacteria, we found three strains 499G2, 499G3, and 499G4 with strong growth-promoting ability. Interestingly, the three strains were all isolated from the rhizosphere of common wild rice. It can be speculated that, among these four species of wild rice, common wild rice has the more helpful rhizosphere microorganisms for the growth of cultivated rice.

In the experimental results, we found that two genera: *Bacillus velezensis* (348Y1, 348Y2, 315Y1, 315Y3, 315Y7, 315G2, and 499G3) and *B. subtilis* (348Y1, 348Y2, 315Y1, 315Y3, 315Y7, 315G2, and 499G3) accounted for a large proportion of the antagonistic bacteria against rice blast. Studies have shown that *B. velezensis* is widely and popularly used as a bacteriostatic agent for various fungal pathogens due to its biocontrol effect [26], for example, Fusarium wilt of cotton, cucumber and peanut, anthracnose of grapes, blight of tomato [27], tobacco browning fungus, tobacco leaf bacteria, wheat scab, rice smut [28], and potato Fusarium wilt, or diseases such as cotton stem blight, cucumber blight, peanut blight, grape anthracnose, tomato late blight, tobacco brown spot, tobacco leaf mold, wheat scab, and potato blight [29,30]. In addition, the strain also has a good growth-promoting effect and can secrete indole acetic acid to promote plant growth [31,32].

### 4.2. Growth-Promoting Effect of Biocontrol Bacteria

Nitrogen, the main component of protein, plays an important role in the growth of stems and leaves and the development of fruits, and is the nutrient element most closely related to plant yield. Chlorophyll is essential for making carbohydrates in plants, and nitrogen is an important part of the synthesis of chlorophyll [33]. Both chlorophyll a and chlorophyll b contain nitrogen compounds, and chlorophyll a content has a stronger correlation with nitrogen than chlorophyll b [34]. Under the action of inoculants, cultivated rice with 499G4 had the highest nitrogen content and chlorophyll content, which coincides with our conclusion.

Phosphorus, as one of the essential elements for plant growth, plays an extremely important role in metabolic processes such as photosynthesis, respiration, and circulation. In agricultural production, it is often one of the limiting factors for high crop yields [35,36]. In our experiment, the total phosphorus content of cultivated rice with inoculum was significantly increased compared with the control. Phosphorus fertilization can promote the normal progress of various metabolisms in the growth and development of plants, and can also improve the stress resistance of plants [37,38].

Potassium can promote the robustness of plant stems, improve fruit quality, enhance plant cold resistance, and increase fruit sugar and vitamin C content. When potassium supply is insufficient, carbohydrate metabolism is disturbed, and photosynthesis is inhibited with enhanced respiration. Therefore, when potassium is deficient, the stress resistance of plants is weakened and the growth of rice seedlings is inhibited [39]. In addition, exogenous potassium application increased the resistance of rice to rice blast. Overexpression of CBL-interacting protein kinase 31 (CIPK31) promoted rice defense to blast, and studies have shown that CIPK31 may interact with AKT1L (a potential K^+^ channel protein) to increase K^+^ uptake, thereby improving resistance to rice blast [40]. 499G3 can significantly increase the potassium content of rice plants, and has a good nitrogen and phosphorus supply capacity, so in addition to effectively preventing rice blast, it also has the highest biomass among the four treatments.

Studies have shown that in leaves pretreated with indole derivatives, the formation of blast spot is inhibited compared to leaves treated with distilled water [41]. Comparing the growth-promoting effect and proficiency test of 499G2 and 499G4, it can be seen that 499G2 promotes the growth of rice by increasing the content of IAA in plants, while 499G4 enhances the photosynthesis of rice to promote the growth of rice. From the application of 499G2 and 499G3, it can be seen that the increase of IAA is more conducive to the growth of plant roots, because 499G3 increases the IAA and chlorophyll of plants at the same time. Therefore, among the four treatments, the recipient plants have the highest stem length and fresh weight. By observing the stem length of the plants, it is found that the stem length of 499G2 is smaller than that of 499G4 and 499G4 significantly affects the chlorophyll level of rice. It can be seen that chlorophylls promote the growth of rice stem. Although the IAA treated by 499G3 was higher than that of 499G2, the increase in root length was not significant, which may be caused by the excessive accumulation of IAA in plants and exceeded its growth-promoting range.

### 4.3. Biocontrol Effect of Biocontrol Bacteria

Carbohydrates are the energy source of cells and the carbon backbone of biosynthetic processes, which have been extensively documented in the literature. In addition, soluble carbohydrates also act as osmotic agents, helping to maintain the integrity of the plasma membrane [42,43], helps plants cope with environmental stress.

Malondialdehyde (MDA) is the product of membrane lipid peroxidation in plant tissues under oxidative stress under adversity. It can cross-link lipids, nucleic acids, carbohydrates and proteins, and react strongly with various components in cells. Reduce the content of unsaturated fatty acids in the membrane, reduce the membrane resistance and membrane fluidity, increase the amount of electrolyte leakage, and change the structure and function of the plasma membrane [44], the accumulation of its content is the manifestation of the toxic effect of free radicals. Therefore, MDA can represent the level of lipid peroxidation of cells and the degree of biofilm damage, reflecting the degree of lipid peroxidation of cell membranes and the strength of plant responses to adverse conditions [45]. Under different stress conditions, the MDA content in rice was significantly increased [46,47]. By comparing the MDA content of blast-resistant and non-blast-resistant rice varieties, it can be clearly seen that the accumulation of MDA in blast-resistant varieties is less [48]. In this experiment, compared with the control, the MDA content of rice seedlings treated with *M. grisea* was significantly increased, which indicated that the cell membrane of the plant had been damaged by oxidation to some extent. The application of microbial inoculum could effectively reduce the accumulation of MDA and help to alleviate the occurrence of membrane lipid peroxidation in rice.

The activities of antioxidant enzymes SOD and POD play an important role in scavenging reactive oxygen species (ROS). SOD plays an important role in protecting cells from oxidative stress. ROS is a strong oxidative compound produced with oxidative metabolism. When faced with environmental stress, ROS and H_2_O_2_ in plants will continue to accumulate, causing great damage to cells [49]. Because it is becomes disproportionate as H_2_O_2_ and O_2_, it exists in cytoplasm and different organelles. Catalytic enzymes eliminate H_2_O_2_ by decomposing H_2_O_2_ into H_2_O and O_2_, and do not need any reduction equivalent [50]. Peroxidase is located in cytoplasm, vacuole, and extracellular space, which can remove H_2_O_2_ by oxidizing substrate. In this experiment, the infection of *M. grisea* significantly increased the activity of antioxidant enzymes in rice seedlings and the effect was more significant after the application of microbial inoculum, which indicated that the addition of microbial inoculum was helpful for rice to remove excessive ROS. It can enhance the defense enzyme activity of rice seedlings and reduce MDA content, thus improving the resistance of rice to rice blast.

## 5. Conclusions

Wild rice is an important improvement resource of cultivated rice. Its unique ability to resist biotic and abiotic stresses has attracted the attention of many scholars. The microbial community structure in rhizosphere and phyllosphere of different rice varieties is also different, which may be one of the reasons for the different stress resistance between wild rice and cultivated rice. In future agricultural development, biological control may become the main trend, and its advantages of green environmental protection and economy will be recognized by more people. However, bacteria with biological control and growth promotion ability just conform to this concept. We screened 45 bacteria isolates from the rhizosphere and phyllosphere of four different wild rice varieties. The results showed that 18 strains had a good inhibitory effect on rice blast, and 33 strains had the ability to dissolve phosphorus, potassium or nitrogen. Through the validation test, the three strains of bacteria (499G2, 499G3, 499G4) not only had resistance to the rice blast, but also had a good effect on the growth of cultivated rice. They increased the content of total nitrogen, total phosphorus, total potassium, IAA, chlorophyll in leaves, and the plant growth. In addition, through the infection verification test of *Magnaporthe grisea*, the application of bacterial agents can significantly reduce the content of MDA, increase the content of soluble sugar, and increase the activity of plant antioxidant enzymes, so as to improve the resistance of rice to *M. grisea*. The discovery of these strains has good prospects for the preparation of inoculants and agricultural applications in the future.

## Figures and Tables

**Table 1:** Bacterial identification results screened from the rhizosphere and phyllosphere of four wild rice species.

Strain Name	Type of Rice	Classification	Part	Starch Hydrolase	IAA Capability (µg/g)
348Y1	*O. punctata*	*Bacillus velezensis*	Shoot	+	−
348Y2	*O. punctata*	*Bacillus velezensis*	Shoot	+	−
348Y3	*O. punctata*	*Bacillus subtilis*	Shoot	+	−
348Y4	*O. punctata*	*Bacillus amyloliquefaciens*	Shoot	+	−
348Y5	*O. punctata*	*Bacillus licheniformis*	Shoot	+	−
348Y6	*O. punctata*	*Bacillus megaterium*	Shoot	−	34.13
348Y7	*O. punctata*	*Fictibacillus nanhaiensis*	Shoot	−	−
348G1	*O. punctata*	*Enterobacter asburiae*	Root	+	47.19
348G2	*O. punctata*	*Brevibacterium frigoritolerans*	Root	−	−
348G3	*O. punctata*	*Bacillus anthracis*	Root	+	−
348G4	*O. punctata*	*Enterobacter asburiae*	Root	−	−
315Y1	*O. officinalis Wall. ex Watt*	*Bacillus velezensis*	Shoot	+	−
315Y2	*O. officinalis Wall. ex Watt*	*Enterobacter asburiae*	Shoot	+	29.29
315Y3	*O. officinalis Wall. ex Watt*	*Bacillus velezensis*	Shoot	+	−
315Y4	*O. officinalis Wall. ex Watt*	*Bacillus simplex*	Shoot	−	26.23
315Y5	*O. officinalis Wall. ex Watt*	*Bacillus thioparans*	Shoot	−	−
315Y6	*O. officinalis Wall. ex Watt*	*Bacillus muralis*	Shoot	+	−
315Y7	*O. officinalis Wall. ex Watt*	*Bacillus velezensis*	Shoot	−	−
315G1	*O. officinalis Wall. ex Watt*	*Bacillus mycoides*	Root	+	−
315G2	*O. officinalis Wall. ex Watt*	*Bacillus velezensis*	Root	+	−
315G3	*O. officinalis Wall. ex Watt*	*Bacillus pseudomycoides*	Root	+	−
315G4	*O. officinalis Wall. ex Watt*	*Bacillus indicus*	Root	−	−
315G5	*O. officinalis Wall. ex Watt*	*Bacillus megaterium*	Root	+	22.52
315G6	*O. officinalis Wall. ex Watt*	*Bacillus methylotrophicus*	Root	−	−
218Y1	*O. nivara*	*Lysinibacillus fusiformis*	Shoot	+	−
218Y2	*O. nivara*	*Bacillus oryzaecorticis*	Shoot	+	−
218Y3	*O. nivara*	*Bacillus subtilis*	Shoot	+	−
218Y4	*O. nivara*	*Bacillus arbutinivorans*	Shoot	−	−
218G1	*O. nivara*	*Pantoea septica*	Root	−	21.87
218G2	*O. nivara*	*Bacillus indicus*	Root	−	−
218G3	*O. nivara*	*Bacillus cereus*	Root	+	−
218G4	*O. nivara*	*Bacillus aryabhattai*	Root	-	121.55
218G6	*O. nivara*	*Bacillus subtilis*	Root	+	−
218G7	*O. nivara*	*Pseudomonas frederiksbergensis*	Root	−	−
499Y1	*O. rufipogon Griff.*	*Bacillus simplex*	Shoot	−	−
499Y3	*O. rufipogon Griff.*	*Bacillus stratosphericus*	Shoot	−	−
499Y5	*O. rufipogon Griff.*	*Bacillus subtilis*	Shoot	+	−
499Y6	*O. rufipogon Griff.*	*Fictibacillus phosphorivorans*	Shoot	−	28.16
499Y7	*O. rufipogon Griff.*	*Bacillus firmus*	Shoot	−	−
499G2	*O. rufipogon Griff.*	*Peribacillus simplex*	Root	+	17.68
499G3	*O. rufipogon Griff.*	*Bacillus velezensis*	Root	+	24.94
499G4	*O. rufipogon Griff.*	*Bacillus megaterium*	Root	+	23.81
499G5	*O. rufipogon Griff.*	*Priestia megaterium*	Root	+	−
499G6	*O. rufipogon Griff.*	*Bacillus anthracis*	Root	+	−
499G7	*O. rufipogon Griff.*	*Fictibacillus phosphorivorans*	Root	+	14.61

‘+’ means to have this ability, ‘−’ means does not have this ability.

**Table 2:** The antagonistic rate of bacterial cells to rice blast plate. Different lowercase letters show statistically significant difference (*p* value < 0.05).

Strain Name	Antagonism Rate (%)	Error Value	Strain Name	Antagonism Rate (%)	Error Value
499G4	87.31 ^a^	0.52	315G1	84.14 ^bcd^	1.47
348Y1	85.98 ^ab^	0.22	315G2	84.07 ^cd^	0.71
315G5	85.28 ^bc^	0.41	499G2	84.07 ^cd^	0.83
315Y3	85.27 ^bc^	0.24	315G6	84.06 ^cd^	1.16
348Y4	84.91 ^bc^	1. 05	499Y5	83.6 ^cd^	0.6
218G6	84.78 ^bc^	1. 39	499G3	82.53 ^de^	1.78
348Y3	84.73 ^bc^	0.28	218Y3	82.39 ^de^	0.99
499Y3	84.72 ^bc^	1. 35	315Y7	81.85 ^e^	0.56
315Y1	84.16 ^bcd^	1. 33	218G1	55.14 ^f^	0.42

**Table 3:** Identification of bacterial growth-promoting ability.

**Potassium-Releasing**			
**Strains Name**	**Effect**	**Strains Name**	**Effect**
218G2	+++	499Y1	+
499Y7	+++	315Y4	+
218G7	+++	499Y5	++
348Y7	++	315G1	+
315G5	++	499Y3	+++
315G2	+++	499G5	++++
218G1	+++	315Y2	++
348Y6	+	499G1	+
348Y4	+	315Y6	++++
499G7	+	348G1	++
348Y5	+	315Y7	+
218Y3	+	348G2	+
315Y1	+	218G4	+++
348Y1	+	499G4	+++
		499G3	+++
**Solubilizing Phosphorus**			
**Strains name**		**Effect**	
348G1		+	
315Y2		+	
218G1		++	
218G7		+	
218G4		+	
**Nitrogen Fixation**			
**Strains name**		**Effect**	
348G1		+++	
348Y7		+++	
315Y7		++	
218G1		+++	
315G5		++	
218G4		++	
218Y3		++	
348Y5		++	

Able to grow in selective medium (+), the transparent circle is the same size as the colony (++), the transparent circle is slightly larger than the colony (+++), the transparent circle is the size of the colony more than 2 times (++++).

## Data Availability

Not applicable.

## Supplementary Materials

The following supporting information can be downloaded at: https://www.mdpi.com/article/10.3390/microorganisms10071468/s1, Figure S1: Pot experiment inoculated with the screened bacteria 499G2, 499G3, and 499G4.
