# Supplementary figures and images for: Culturable Screening of Plant Growth-Promoting and Biocontrol Bacteria in the Rhizosphere and Phyllosphere of Wild Rice
Source: Microorganisms. 2022 Jul 20;10(7):1468. doi: 10.3390/microorganisms10071468 (PMC9324538; doi:10.3390/microorganisms10071468)

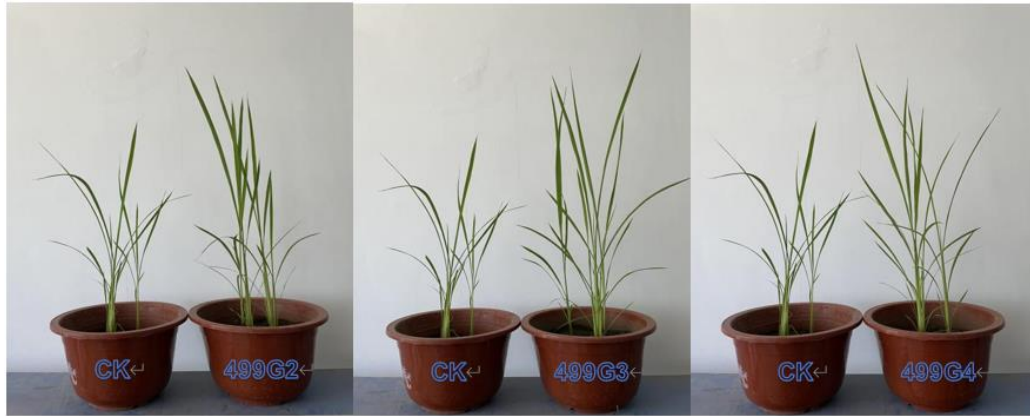

**Figure S1.** Pot experiment inoculated with the screened bacteria 499G2, 499G3, and 499G4.

Supplement: Supplementary file 1 [file microorganisms-10-01468-s001.zip › microorganisms-1777591-supplementary.pdf]
